# The spectrum of heart disease among adults at the Bamenda Regional Hospital, North west Cameroon: a semi urban setting

**DOI:** 10.1186/s13104-019-4803-1

**Published:** 2019-11-21

**Authors:** Manuel Ndo Akono, Larissa Pone Simo, Valirie Ndip Agbor, Sylvain Laah Njoyo, Dora Mbanya

**Affiliations:** 1 Bamenda Regional Hospital, Bamenda, Cameroon; 2 Faculty of Health Sciences, The University of Bamenda, Bamenda, Cameroon; 3 Nuffield Department of Population Health, University of Oxford, Oxford, UK; 4 Department of Health Research, Health Education and Research Organization (HERO), Buea, Cameroon; 5 Mozogo Sub-divisional Hospital, Mayo-Moskota, Far North Region, Mozogo, Cameroon; 6 Yaoundé University Teaching Hospital (YUTH), Yaoundé, Cameroon

**Keywords:** Heart disease, Aetiologies, Mixed cardiopathies, Heart failure, Bamenda, Cameroon

## Abstract

**Objective:**

We sought to determine the spectrum of heart diseases among adult patients at the Bamenda Regional Hospital in the North West Region of Cameroon. This is a chart review of echocardiographic records.

**Results:**

In total, 673 records were included in our study, of which 506 had a definite heart disease. Of the 506, 93 had mixed cardiopathies. Their ages ranged from 18 to 105 years with a median age of 64.0 (Interquartile range = 47–75) years. Females accounted for a greater proportion (55.3%) of the study population. The most common echocardiographic diagnoses were hypertensive heart disease (41.1%), valvular heart disease (22.3%) and cardiomyopathies (11.4%). The prevalence of heart failure was 17.5%, with hypertensive heart disease being the leading cause.

## Introduction

The exponential rise in the use of antibiotics and vaccines, and increase in cardiovascular risk factors like hypertension and diabetes owing to westernisation and urbanisation are creating a rapid shift in the epidemiological transition from communicable to non-communicable diseases (NCDs) in sub-Saharan Africa (SSA) [1]. Non-communicable diseases are the major cause of mortality worldwide and are expected to be responsible for about 69% of the global mortality rate by 2030 [2]. Cardiovascular diseases are the second most common cause of death in SSA, after the Human Immunodeficiency Virus/Acquired Immune Deficiency Syndrome (HIV/AIDS) [3–5]. In Cameroon, NCDs are estimated to account for 31% of all deaths, 14% of which are due to cardiovascular diseases [3].

Heart failure is associated with a 360-day all-cause mortality rate of 21.9–51.9% in SSA, and is the carrefour for a greater majority of poorly diagnosed or managed heart diseases [6]. As at now, non-ischemic heart diseases are known to be the most frequent causes of heart failure in SSA, with rheumatic heart disease (RHD), hypertensive heart disease (HHD) and non-ischemic cardiomyopathies (CMO) accounting for over 75% of cases [4, 7, 8].

In Cameroon, there is a dearth of evidence on the profile of heart diseases with a few hospital-based studies in urban, semi-urban and rural areas of the country [9–11]. These studies highlight HHD, cardiomyopathy and RHD as the leading causes of heart disease in Cameroon. In a bit to add to the body of knowledge on the subject in Cameroon and SSA, we sought to determine the pattern of heart diseases among adult patients received for echocardiography in a referral hospital in the Northwest Region of Cameroon.

## Main text

### Methods

#### Study design, duration, setting and participants

We carried out a descriptive and retrospective study of echocardiographic records of patients aged 18 years or older referred for echocardiography from July 2015 to April 2018 at the Imagery Centre of the Bamenda Regional Hospital, Bamenda. The Bamenda Regional Hospital is the main referral government-owned hospital in the Northwest Region of Cameroon, and also acts as a teaching hospital for the Faculty of Health Sciences of the University of Bamenda, Cameroon. Records of patients below 18 years old and repeat procedures were excluded.

#### Data collection and sampling

Ethical clearance and administrative authorisation were obtained prior to the study. Transthoracic bi-dimensional M mode coupled with Doppler echocardiographic examinations were performed with a HITACHI F37 echography instrument and a 4–7 Megahertz transducer. Measurements and diagnoses were based on the recommendations of the American Society of Echocardiography (ASE) [12].

Data collected included the age, gender, comorbidity(ies), left ventricular ejection fraction and echocardiographic diagnosis. Records were exhaustively included using a consecutive sampling.

#### Data management and statistical analysis

Data was entered into Epidata version 3.1 and analysed using SPSS version 20.0. Medians and interquartile ranges were used to summarise continuous variables, while proportions, frequencies and charts were used to describe categorical variables. Differences in proportions were compared using the Bonferroni’s test. p values < 0.05 were considered statistically significant.

### Results

In total, 719 echocardiograms were performed during the study period. Forty-six records were excluded for incomplete data; and a total of 673 records were included for data analysis. Of these, 506 had a definite heart disease. Amongst those with a definite heart disease, 93 (13.9%) had mixed cardiopathies. The ages of the participants ranged from 18 to 105 years, with a median age of 64.0 (Interquartile range [IQR] = 47–75) years. Females accounted for 55.3% of the study population. The most common comorbidity was hypertension (51.7%) followed by stroke (4.9%) and HIV (2.5%).

Valvular heart disease was the major cause of heart disease among young adults < 30 years 10 (18.9%), while HHD was consistently the commonest cause of heart diseases among participants who were 30-years and old. Also, there was an increasing trend of mixed heart disease increases with age (Table 1).

**Table 1:** Echocardiographic diagnosis by age group, Bamenda Regional Hospital, July 2015 to April 2018

Diagnosis	Age groups (in years)							
	[< 30] N (%)	[30–39] N (%)	[40–49] N (%)	[50–59] N (%)	[60–69] N (%)	[70–79] N (%)	[≥ 80] N (%)	Total (%) N = 673
Hypertensive HD	3 (5.7)	7 (12.3)	22 (29.7)	28 (28.0)	44 (33.6)	61 (37.2)	27 (29.3)	192 (28.5)
Cor Pulmonale	1 (1.9)	5 (8.8)	6 (8.1)	2 (2.0)	8 (6.1)	10 (6.1)	4 (4.3)	36 (5.3)
Cardiomyopathy	3 (5.7)	4 (7.0)	0 (0.0)	8 (8.0)	12 (9.2)	12 (7.3)	7 (7.6)	46 (6.8)
Valvular HD	10 (18.9)	6 (10.4)	10 (13.5)	10 (10.0)	12 (9.2)	16 (9.8)	15 (16.3)	79 (11.7)
Congenital HD	1 (1.9)	0 (0.0)	0 (0.0)	0 (0.0)	0 (0.0)	0 (0.0)	0 (0.0)	1 (0.2)
Ischaemic HD	0 (0.0)	0 (0.0)	0 (0.0)	4 (4.0)	5 (3.8)	6 (3.7)	5 (5.5)	20 (3.0)
Pericardial Dz	8 (15.1)	7 (12.3)	4 (5.4)	3 (3.0)	1 (0.8)	3 (1.8)	2 (2.2)	28 (4.2)
Mixed HD	5 (9.4)	7 (12.3)	6 (8.1)	14 (14.0)	19 (14.5)	21 (12.8)	21 (22.8)	93 (13.9)
Others	0 (0.0)	1 (1.8)	1 (1.4)	3 (3.0)	2 (1.5)	1 (0.6)	1 (1.1)	11 (1.6)
Normal exam	22 (41.5)	20 (35.1)	25 (33.8)	28 (28.0)	28 (21.3)	34 (20.7)	10 (10.9)	167 (24.8)
Total	53 (100)	57 (100)	74 (100)	100 (100)	131 (100)	164 (100)	92 (100)	673 (100)

*N* frequency, *HD* heart disease, *Dz* diseases

Of the 506 echocardiographic records with a heart disease, a total of 615 individual cases of heart diseases were identified (including mixed heart diseases). Hypertensive heart disease (41.1%), VHD (22.3%) and CMO (11.4%) were the commonest heart diseases identified. Ischaemic heart disease was rare (7.0%), Table 2. Males were more than twice as likely to have an ischaemic heart disease compared to their female counterparts (10.4% versus 3.8%; p < 0.001), and vice versa for pericardial diseases (9.8% versus 6.1%; p = 0.046), Table 2.

**Table 2:** Proportion of different heart diseases (excluding normal cases)

Diagnosis	Total (N = 615)		Male (N = 298)		Female (N = 317)		*p*-value
	Frequency	Percentage	Frequency	Percentage	Frequency	Percentage	
Hypertensive HD	253	41.1	122	40.9	131	41.3	0.200
Ischaemic heart disease	43	7.0	31	10.4	12	3.8	< 0.001*
Cor Pulmonale (CP)	51	8.3	23	7.7	28	8.8	0.973
Acute CP	13	25.5	5	21.8	8	28.6	
Chronic CP	20	39.2	9	39.1	11	39.3	
Primary Pulmonary Hypertension	18	35.3	9	39.1	9	32.1	
Cardiomyopathy	70	11.4	39	13.1	31	9.8	0.054
Hypertrophic	15	21.4	6	15.4	9	29.0	
Dilated	54	77.2	33	84.6	21	67.7	
Myocarditis	1	1.4	0	0	1	3.3	
Valvular heart disease	137	22.3	57	19.2	80	25.2	0.389
Rheumatic heart disease	8	5.8	1	1.8	7	8.8	
Aortic insufficiency	28	20.4	19	33.3	9	11.3	
Aortic stenosis	22	16.1	10	17.5	12	15.0	
Mitral insufficiency	63	46.0	19	33.3	44	55.0	
Mitral stenosis	2	1.5	1	1.8	1	1.2	
Pulmonary insufficiency	1	0.7	0		1	1.2	
Pulmonary stenosis	1	0.7	1	1.8	0	0.0	
Polyvalvulopathy	12	8.8	6	10.5	6	7.5	
Congenital HD	1	0.2	1	0.3	0	0.0	–
Sinus venosus	1	100.0	1	100.0	0	0.0	
Pericardial Disease	49	8.0	18	6.1	31	9.8	0.046*
Pericarditis	18	36.7	8	44.4	10	32.3	
Effusive pericariditis	30	61.3	10	55.5	21	67.7	
Constrictive	1	2.0	1	0.1	0	0.0	
Others	11	1.8	7	2.3	4	1.3	0.066
Aortic aneurysm	8	72.7	6	85.7	2	50.0	
Isolated dilatation of LA	1	9.1	1	14.3	0	0.0	
Bi-auricular dilatation	2	18.2	0	0.0	2	50.0	

*HD* heart disease, *CP* cor pulmonale, *LA* left atrium

**Fig. 1:**
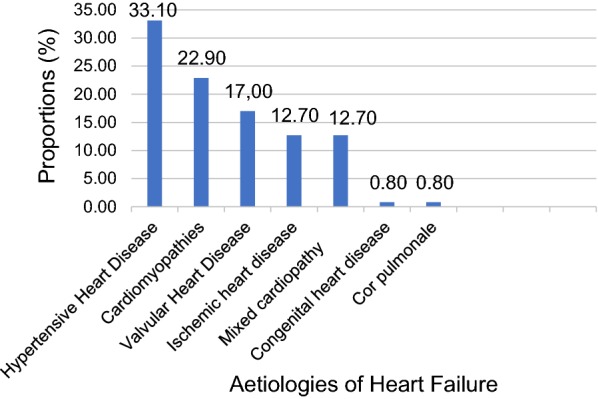
Aetiologies of heart failure with reduced ejection fraction

Heart failure with reduced ejection fraction (HFrEF) occurred in 118 (17.5%) patients, the Median age was = 65.0 years, with patients aged over 70 years bearing the greatest burden and slightly over half of the heart failure cases occurring in males 63 (53.4%). Hypertensive heart disease was the leading cause Fig. 1.

### Discussion

We sought to describe the profile of heart diseases among adult patients referred for echocardiography in a semi-urban setting of Cameroon. Hypertension accounted for over half of the comorbidity among these patients. Hypertension remains a driver of cardiovascular diseases burden in SSA [5, 13, 14].

Hypertensive heart disease was the leading type of heart disease in our setting, accounting for 41.1%. This is similar to findings of Tchoumi and Bureta conducted in a rural setting of the Northwest Region [11] and other semi-urban and urban settings in Cameroon [9–11, 15]. Given the high hypertension rates in the general Cameroonian population [16], coupled with the contrastingly poor awareness, treatment and control rates [16–19], our findings were expected. Similar findings have been reported in other SSA series [7, 20, 21]. However, our results differ from studies conducted in East Africa. For example, in an Ethiopia study, valvular heart disease was the commonest heart disease [22]. In addition, pericardial diseases were the main types of heart disease in a Malawian study [23]. This discrepancy could be due to the relatively younger population in the Ethiopian study [22]. Indeed, rheumatic heart disease which is a significant cause of VHD in SSA is a disease of children and adolescents [24]. An early diagnosis of hypertension, treatment and, most importantly, close follow up on treatment could significantly delay the occurrence of hypertensive heart disease in our setting. In addition, Of the 93 patients who presented with mixed cardiopathies, hypertensive heart disease associated mixed cardiopathies were still the most common 61 (65.6%).

Valvular heart disease was the second most common type of heart disease in our setting. It accounted for 11.7% of the study population, and of these only 5.8% was secondary to rheumatic heart disease. More than half of reported cases were related to degenerative processes. Our findings are contrary to those of Jingi et al. and Nkoke et al. of the West and Southwest regions of Cameroon respectively, who reported cardiomyopathies as the second leading type of heart disease [9, 10]. Even though rheumatic heart disease remains a major public health issue in Cameroon and SSA as a whole, it seems to be relatively scarce in our study setting. This could be explained by the fact that rheumatic heart disease occurs in a relatively young population and people aged < 30 years represented only 8.3% of our study population. Nonetheless, 1.1% of all cases of heart disease was secondary to RHD in our study, which is relatively lower than 3.4% reported by Jingi et al. in the West Region [10].

The relatively lower proportion of post rheumatic valvulopathies could also be explained by the fact that being a hospital-based study, cases which were not clinically evident and those who could not afford a cardiac ultrasound did not feature in the study. In addition, the fact that there are no available internationally standardised guidelines for the diagnosis of RHD in adults [25] might have contributed to an underestimation of the true prevalence of the disease in this study, especially as over 90% of our study population was at least 30 years old.

The prevalence of cardiomyopathies was 6.8%, and they were the third commonest type of heart disease in our setting. Dilated cardiomyopathy remained the most common type of cardiomyopathy, accounting for 77.2% of cardiomyopathies. Our findings differ from those reported in the South West and West regions of the Cameroon [9, 10] in that cardiomyopathies were the third and not second most frequent type of heart disease in our setting. Though dilated cardiomyopathies were still the major types of cardiomyopathies, hypertrophic cardiomyopathies were the second most common cardiomyopathies with a proportion of 21.4%.

The spectrum of pericardial disease in our study is relatively lower than that reported by Jingi et al. in West Cameroon and relatively higher than that reported by Nkoke et al. in the South West Cameroon [9, 10]. However, findings in Malawi report pericardial diseases as the leading burden of heart disease; associating it to the coinciding HIV epidemic. The most common type of pericardial disease in our context was the pericarditis with effusion, of which 20% were associated to tuberculosis.

It is worth noting that up to 13.9% of our study population was diagnosed with more than one heart disease, what we termed mixed cardiopathies. To the best of our knowledge, this is the first study in Cameroon which reports a population with patients having more than one heart disease. This is important knowledge necessary in the diagnosis, management and prognosis of heart diseases in our context.

Heart failure occurred in 17.5% of study population, with HHD (33.1%), CMO (22.9%) and VHD (17%) responsible for over 70% of the cases. Mixed cardiopathies significantly contributed to HF in our study.

The proportionately more males with heart failure in our study is similar to that reported by Kingue et al. in the General Hospital of Yaoundé, Cameroon [26]. However, there is a gender disparity between our study and findings in the Heart of Soweto study in South Africa and the Abuja Heart study in Nigeria where females formed the greater proportion of the population with heart failure [27, 28].

According to a recent review, HF in sub-Saharan Africa was reported as a disease of the middle-aged adult, occurring between the third and fifth decades of life, as opposed to the developed world where it is essentially a disease of the elderly, occurring in the seventh decade of life [6]. In addition, HHD, CMO and VHD were the leading causes of HF [6]. Though our findings still maintain HHD as the leading cause of heart failure in our study population, they differ from existing knowledge in that valvular heart diseases are the third commonest causes [29]. Given that hypertensive heart disease arises as a complication of longstanding systemic hypertension on the heart, with its increasing prevalence in SSA, the importance of early prevention and proper treatment of hypertension in our context cannot be over emphasized. Rheumatic heart disease instead was a rare cause of heart failure here probably accounted for by the age limit of our population. Nonetheless, mixed cardiopathies formed a sizeable proportion of the aetiologies of heart failure in our context.

### Conclusion

Hypertensive heart disease is the most common heart disease in this semi-urban setting in Cameroon. A sizeable proportion of the population has more than one heart disease (mixed cardiopathies). Prevention of heart diseases in our setting should re-enforce awareness, mass screening, and the treatment and control of hypertension. Rendering cardiac ultrasound more available and affordable, might go a long way to improve the diagnosis, treatment and follow up of patients with heart diseases in the North West Region, and as a result, reduce the incidence of heart failure in our context.

## Limitation

The retrospective design of our study gave us no control on the quality of the echocardiographic records. The diagnosis of certain cardiopathies could have been wrongly classified due the absence of records of other investigations. For example, some cases of ischaemic dilated cardiomyopathy might have been classified as non-ischemic due to absence of recorded of further investigations such as electrocardiography, stress test or coronary angiography. Furthermore, we recorded a low prevalence of rheumatic valve diseases due to difficulties of the resourceless population to get to the hospital and afford payment for echocardiography. However, with strict selection criteria, we hope this reflects to a certain degree the spectrum of heart disease among patients referred for echocardiography at the Bamenda Regional Hospital.

## Data Availability

The datasets for this study are available from the corresponding author on reasonable request.

## Abbreviations

ASE: American Society of Echocardiography
CMO: cardiomyopathy
HF: heart failure
HFrEF: heart failure with reduced ejection fraction
IQR: interquartile range
NCDs: non-communicable diseases
RHD: rheumatic heart disease
SPSS: statistical package for social sciences
SSA: sub-Saharan Africa
VHD: valvular heart disease
